# Elevated blood viscosity is associated with cerebral small vessel disease in patients with acute ischemic stroke

**DOI:** 10.1186/s12883-017-0808-3

**Published:** 2017-01-31

**Authors:** Seung Hoon Song, Jeong Hee Kim, Joon Hwa Lee, Yeo-Min Yun, Dong-Hee Choi, Hahn Young Kim

**Affiliations:** 1 Department of Neurology, Research Institute of Medical Science, Konkuk University School of Medicine, Konkuk University Medical Center, Seoul, Republic of Korea; 2 Department of Laboratory Medicine, Konkuk University School of Medicine, Konkuk University Medical Center, Seoul, Republic of Korea; 3 Department of Medical Science, Konkuk University School of Medicine, Seoul, Republic of Korea

**Keywords:** Blood viscosity, Small vessel disease, Small artery occlusion, Lacunes, Ischemic stroke

## Abstract

**Background:**

Increased level of blood viscosity, which is one of the major factors that determine blood rheology, has been reported as a risk factor or predictor for cerebrovascular events. We investigated how blood viscosity is associated with acute stroke and chronic radiological manifestations of cerebral small vessel disease, and how blood viscosity changes after stroke.

**Methods:**

We prospectively enrolled consecutive patients with acute ischemic stroke. Whole blood viscosities at a low or high shear rate were measured using a scanning capillary tube viscometer, and were referred to as diastolic blood viscosity (DBV) and systolic blood viscosity (SBV), respectively. Correlations between blood viscosity and acute stroke etiology or chronic radiological manifestations of cerebral small vessel disease were investigated. The temporal profiles of blood viscosity at the onset of stroke and follow-up at 1 and 5 weeks were investigated.

**Results:**

Of the 127 patients admitted with acute ischemic stroke, 63 patients were included in the final analyses. DBV at the onset of stroke was significantly higher in small artery occlusion (SAO) stroke than in other stroke subtypes (*p* = 0.037). DBV showed a significant positive correlation with the number of chronic lacunes (*r* = 0.274, *p* = 0.030). The temporal profiles of DBV in SAO stroke showed a transient decrease due to the hydration therapy after 1 week and recurrent elevation at 5 week follow-up (*p* = 0.009).

**Conclusions:**

Our study suggests that elevated DBV may play a role in the development of acute and chronic manifestations of cerebral small vessel disease. The recurring elevation of DBV in SAO stroke indicates that sufficient hydration and additional therapeutic interventions targeting blood viscosity may be needed in patients with SAO stroke.

## Background

Increased fibrinogen level, whole blood viscosity, and plasma viscosity, which are major factors that determine blood rheology, have been reported as risk factors or predictors for various cardio- or cerebrovascular events [1–11]. Whole blood viscosity can be determined through the complex interactions of blood constituents, including red blood cells, white blood cells, platelets, fibrinogen, and other plasma proteins [12, 13]. Whole blood viscosity measured at a high shear rate of 300 s^−1^ or more is referred to as systolic blood viscosity (SBV) [14, 15]. Blood viscosity at a high shear rate represents the frictional characteristics of fast blood flow, defined as having a velocity of over 30 cm/s in a vessel with a large diameter [13]. In the high blood flow state, the inertia force representing the kinetic energy is much larger than the viscous force that arises from the blood viscosity [15]. Therefore, blood flow is predominantly determined by the inertia force rather than the viscous force [15]. Meanwhile, the whole blood viscosity measured at a low shear rate of 5 s^−1^ or less is referred to as diastolic blood viscosity (DBV) [14, 15]. In the low blood flow state, in which the blood flows through a vessel with a smaller diameter or through the distal part of an occluded vascular segment, blood viscosity becomes a critical factor for determining the tissue perfusion status [13]. Therefore, DBV may be attributed more to the pathophysiology of small vessel disease than SBV. In the case of coronary microvascular dysfunction without definite occlusion of the main coronary artery, i.e. microvascular angina, increased blood viscosity may play a key role in the deterioration of tissue perfusion at a microvascular level [16]. A comparable microvascular dysfunction in the brain may be chronic cerebral ischemia due to cerebral small vessel disease [17, 18]. Radiological manifestations, such as white matter hyperintensities, lacunes, and microbleeds, are known consequences of impaired microvascular perfusion in the brain and chronic radiological markers for cerebral small vessel disease [19, 20].

The role of blood viscosity has been reported in the stroke population [1–7]. Previous studies, which included stroke subtype in the scope of their studies, have suggested that the blood viscosity measured in small artery occlusion (SAO) stroke was higher than in other stroke subtypes, such as large artery atherosclerosis (LAA) and/or cardioembolism (CE) [2–4]. Based on these previous studies, we hypothesized that blood viscosity may be closely associated with acute or chronic manifestations of cerebral small vessel disease. We first questioned how blood viscosity is related to different etiologies leading to acute stroke. Second, we asked how blood viscosity is associated with the chronic radiological manifestations of cerebral small vessel disease. Third, we asked if there is an abnormality in blood viscosity in relation to acute stroke, how it changes after acute stroke. For the first two questions, we investigated which stroke subtype at the onset of index stroke and which chronic radiological manifestations, including white matter hyperintensities, lacunes, and microbleeds, are correlated with blood viscosity in patients with acute ischemic stroke. For the last question, the temporal profiles of blood viscosity at both stroke onset and follow-up were investigated.

## Methods

### Patients and blood sampling

We prospectively enrolled consecutive acute ischemic stroke patients who had been admitted within 3 days of the onset of stroke, from July 2013 to February 2014. Acute ischemic stroke was confirmed by diffusion-weighted magnetic resonance imaging (MRI) in all patients. To minimize the diluting effect on blood viscosity by hydration therapy, the blood samples used to measure the baseline blood viscosity levels were obtained during the initial blood sampling procedure in the emergency room prior to any medical intervention including hydration therapy. Follow-up measurements of blood viscosity were performed twice, at 1 week and 5 weeks after the baseline measurement. To minimize the transient diluting effect by oral hydration, the samples for the follow-up measurement were obtained under fasting state. Patients were instructed not to eat or drink anything, including water, after taking their evening pills until blood sampling was performed early the next morning. Blood samples from patients diagnosed with stroke mimic during the same study period were used with permission, for comparison with those from patients with acute ischemic stroke. This study was approved by the institutional review board of the Konkuk University Hospital. Written informed consent was obtained from each patient.

### Clinical evaluation

The vascular risk factors and laboratory findings affecting blood viscosity including hemoglobin, hematocrit, white blood cells, platelets, blood protein, fibrinogen, D-dimer, BUN/Cr (blood urea nitrogen-to-creatinine) ratio, and lipid profiles were investigated. The patients were considered to have hypertension if they were on medication for hypertension or had a blood pressure of ≥140/90 mmHg on repeated measurement. Similarly, the patients were considered to have diabetes mellitus if they were on medication for diabetes or had a fasting blood sugar ≥126 mg/dl; and to have hyperlipidemia if they were on lipid-lowering medications or had an overnight fasting cholesterol level ≥220 mg/dl or low-density lipoprotein cholesterol level ≥140 mg/dl. Smoking was defined by the current smoking status. The stroke subtype was classified according to the modified Trial of Org 10172 in Acute Stroke Treatment classification [21].

### Blood viscosity measurement

A scanning capillary tube viscometer (BVD-PR01, Bio-Visco Inc., Korea) was used to measure whole blood viscosity [15]. The scanning capillary tube viscometer simultaneously measures the SBV and DBV, which correspond to the whole blood viscosities at high and low shear rates, respectively [15]. Samples containing 3 mL whole blood were collected in a vacutainer containing ethylenediaminetetraacetic acid (EDTA) anticoagulant and stored in a refrigerator at 4 °C until measurement. All the measurements were performed within 24 h of the collection. The whole blood viscosity was measured over a wide range of shear rates, ranging from 1 to 1000 s^−1^. In the low shear rate range of 5 s^−1^ or less, a whole blood viscosity level of 1 s^−1^ was selected as the DBV for our study. In the high shear rate range of 300 s^−1^ or more, a whole blood viscosity level of 300 s^−1^ was selected as the SBV for our study.

### Radiological evaluation

T1-weighted, T2-weighted, fluid attenuation inversion recovery (FLAIR), and T2* gradient-echo images were obtained using a 3 T MR system (Signa HDx; GE Medical Systems, Milwaukee, WI, USA). The T1- and T2-weighted images were obtained during the same imaging session at the same orientation and slice position: matrix, 128; field of view, 280 × 280 mm; section thickness, 5 mm; intersection space, 2 mm. The radiological manifestations associated with small vessel disease in the brain, such as white matter hyperintensities, lacunes, and microbleeds were subsequently defined. The severity of white matter hyperintensities on FLAIR images were assessed by a scale of periventricular hyperintensity (PVH) and deep white matter hyperintensity (DWMH) according to the Fazekas scale [22]. Lacunes were distinguished from white matter ischemic changes as small distinct lesions (<15 mm in diameter) with a low signal on T1-weighted images, a high signal on T2-weighted images, and perilesional gliotic changes on FLAIR images [23]. Lacunes usually had a hyperintense rim around the cavity on FLAIR images, unlike Virchow Robin spaces or white matter ischemic changes. Acute lacunar infarctions that presented as an index stroke when enrolling patients, were not included in the analysis. Only previous chronic lacunes were included, regardless of whether they were symptomatic or asymptomatic. A microbleed was defined as a small, homogenous, round signal loss lesion (<10 mm in diameter) on T2* gradient-echo images [23].

### Statistical analysis

The differences in vascular risk factors or laboratory findings were assessed using a chi square test for dichotomized variables, and either an independent *t* test, Mann-Whitney *U* test, or analysis of variance (ANOVA) with a Tukey’s post-hoc test for continuous variables, where appropriate. The association between SAO stroke and the laboratory variables was assessed using a calculation of crude or adjusted odds ratios with 95% confidence intervals through uni- or multivariate logistic models. The associations among the laboratory findings, including DBV, SBV, hematocrit, and fibrinogen levels were analyzed using a Pearson correlation. The associations between laboratory and radiological findings were also analyzed using a Pearson correlation. Differences were considered statistically significant at *p* values of < 0.05. All statistical analyses were performed using SPSS version 17.0.

## Results

### Patient enrollment

**Fig. 1:**
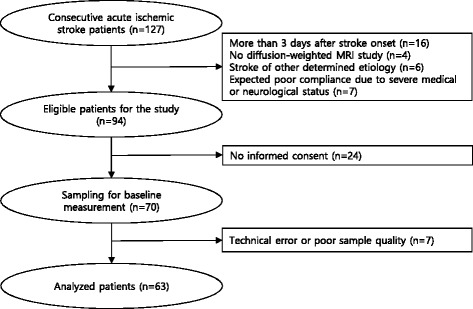
Patient enrollment flow chart

Of the 127 consecutive patients with acute ischemic stroke admitted during the study period from July 2013 to February 2014, samples for blood viscosity measurement were obtained from 70 patients (Fig. 1). In order to homogenize the study population, 16 patients with a stroke onset of more than 3 days, 4 patients whose stroke was not confirmed by diffusion-weighted MRI, 6 patients with stroke of other determined etiology, and 7 patients with severe medical or neurological status were excluded. Of the 94 patients eligible for the study, 24 patients refused to provide written informed consent. Seven samples were discarded due to either technical errors or poor quality. Thus, data from 63 patients were analyzed in our study. Sixteen patients were included in the stroke mimic group, which was comprised of 7 patients with non-specific dizziness, 5 patients with transient weakness without a vascular cause, 3 patients with vestibular neuronitis, and 1 patient with hypochondriasis. Patients with suspected transient ischemic attack (TIA) were not included in the stroke mimic group because TIA can be also influenced by blood viscosity.

### Demographic and clinical characteristics of patients

The clinical demographics, radiological findings, and laboratory findings of patients are presented in Table 1. The most frequent stroke subtype was SAO (*n* = 25, 39.7%) followed by LAA (*n* = 23, 36.5%), CE (*n* = 12, 19.0%), and cryptogenic stroke (Cryp; *n* = 3, 4.8%). The prevalence of vascular risk factors, except for atrial fibrillation, were not different among the stroke subtypes (Table 1).

**Table 1:** Clinical demographics, radiological findings, and laboratory findings for the 63 patients included in the study

	Total (*n* = 63)				
	SAO (*n* = 25, 39.7%)	LAA (*n* = 23, 36.5%)	CE (*n* = 12, 19.0%)	Cryp (*n* = 3, 4.8%)	*P* value
Blood viscosity (mean ± sd)					
Diastolic blood viscosity, mP	274.7 ± 70.9	214.5 ± 75.9	252.0 ± 57.5	258.9 ± 46.5	0.037*
Systolic blood viscosity, mP	45.2 ± 16.0	38.1 ± 12.4	40.0 ± 4.9	39.0 ± 4.7	0.273
Clinical findings					
Age, years (mean ± sd)	62.7 ± 12.9	67.0 ± 15.5	71.2 ± 11.6	63.3 ± 7.5	0.335
Male, *n* (%)	19 (76)	13 (57)	7 (58)	2 (67)	0.513
Hypertension, *n* (%)	16 (64)	16 (70)	5 (42)	3 (100)	0.207
Diabetes, *n* (%)	6 (24)	7 (30)	3 (25)	0 (0)	0.716
Hyperlipidemia, *n* (%)	6 (24)	3 (13)	1 (8)	0 (0)	0.484
Atrial fibrillation, *n* (%)	0 (0)	0 (0)	9 (75)	0 (0)	<0.001
Smoking, *n* (%)	14 (56)	8 (35)	2 (17)	0 (0)	0.053
Radiological findings, median (IQR)					
PVH, Fazekas scale	1 (1–2)	1 (1–2)	1 (1–3)	2 (0–2)	0.872
DWMH, Fazekas scale	1 (0–1.5)	1 (0–2)	1 (0–2.75)	1 (0–1)	0.834
Number of chronic lacunes	1 (0–3)	0 (0–2)	1 (0–2.75)	1 (0–2)	0.255
Number of microbleeds	0 (0–2.5)	0 (0–1)	1 (0–2)	0 (0–2)	0.869
Laboratory findings (mean ± sd)					
Hemoglobin, g/dL	14.9 ± 1.8	13.9 ± 1.9	13.6 ± 1.7	13.7 ± 1.9	0.129
Hematocrit, %	43.4 ± 5.2	40.9 ± 5.0	39.8 ± 4.4	40.0 ± 5.0	0.135
White blood cells, 10^3^/μL	7.2 ± 2.0	7.6 ± 2.9	9.5 ± 3.6	6.8 ± 2.6	0.110
Platelets, 10^3^/μL	238 ± 56	227 ± 48	233 ± 80	199 ± 40	0.700
Total protein, gm/dL	7.1 ± 0.5	7.0 ± 0.4	7.2 ± 0.5	7.2 ± 0.1	0.429
Fibrinogen, mg/dL	323 ± 78	335 ± 71	357 ± 70	215 ± 44	0.099
D-dimer, μg/dL	0.44 ± 0.32	0.75 ± 0.92	1.51 ± 1.93	0.28 ± 0.10	0.038**
BUN/Cr ratio	19.0 ± 4.6	23.1 ± 8.2	21.1 ± 5.3	17.3 ± 5.1	0.115
Total cholesterol, mg/dL	176 ± 36	177 ± 28	155 ± 56	140 ± 32	0.192
Low-density lipoprotein, mg/dL	109 ± 32	108 ± 22	94 ± 50	80 ± 30	0.319
Triglycerides, mg/dL	127 ± 61	116 ± 80	70 ± 60	60 ± 35	0.078

*Abbreviations*: *SAO* small artery occlusion, *LAA* large artery atherosclerosis, *CE* cardioembolism, *Cryp* cryptogenic stroke, *sd* standard deviation, *IQR* interquartile range, *PVH* periventricular hyperintensity, *DWMH* deep white matter hyperintensity, *BUN/Cr* blood urea nitrogen-to-creatinine

*, *p* = 0.021, SAO vs. LAA in the post-hoc test; **, *p* = 0.027, SAO vs. CE in the post-hoc test

### Blood viscosity, hematocrit, and blood fibrinogen level

**Fig. 2:**
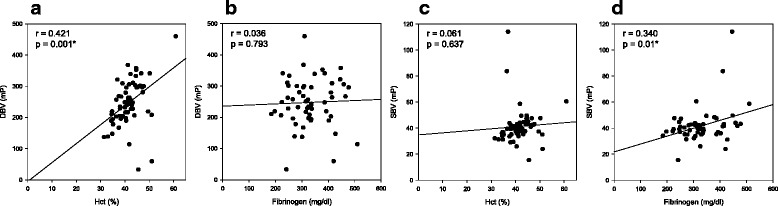
The correlation of diastolic blood viscosity with hematocrit **a** and blood fibrinogen level **b**. The correlation of systolic blood viscosity with hematocrit **c** and blood fibrinogen level **d**. *, *p* < 0.05; *DBV*, diastolic blood viscosity; *SBV*, systolic blood viscosity; *Hct*, hematocrit

The DBV and SBV were significantly correlated with each other (*r* = 0.456, *p* < 0.001). The DBV was significantly correlated with hematocrit (*r* = 0.421, *p* = 0.001; Fig. 2a) and the SBV with blood fibrinogen level (*r* = 0.340, *p* = 0.01; Fig. 2d).

### Blood viscosity and stroke subtypes

**Fig. 3:**
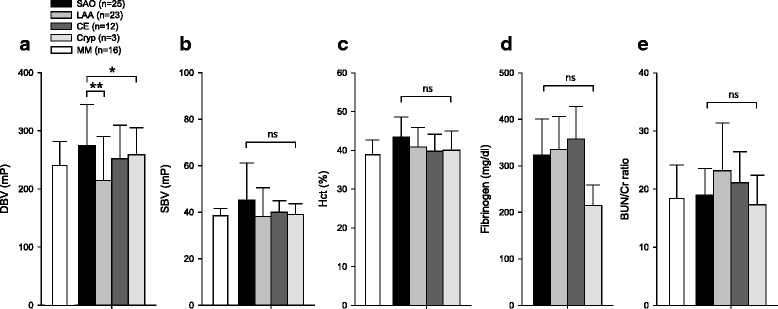
Blood viscosity and stroke subtypes. The diastolic blood viscosity was highest in patients with SAO stroke **a**. The systolic blood viscosity, hematocrit, blood fibrinogen level, and BUN/Cr ratio were not different among the stroke subtypes **b**, **c**, **d**, and **e**. *p < 0.05 on ANOVA; **p < 0.05 on the post-hoc test; ns, not significant; *SAO*, small artery occlusion; *LAA*, large artery atherosclerosis; *CE*, cardioembolism; *Cryp*, cryptogenic stroke; *MM*, stroke mimic; *DBV*, diastolic blood viscosity; *SBV*, systolic blood viscosity; *Hct*, hematocrit; *BUN*, blood urea nitrogen

The baseline DBV was 274.7 ± 70.9 mP in SAO, followed by 258.9 ± 46.5 mP in Cryp, 252.0 ± 57.5 mP in CE, and 214.5 ± 75.9 mP in LAA stroke subtypes. The baseline DBV in SAO was significantly higher than that in other stroke subtypes (*p* = 0.037). In addition, the post-hoc test revealed that the baseline DBV in SAO was significantly higher compared to that of LAA (*p* = 0.021; Table 1 and Fig. 3a). Among laboratory variables which were significantly associated with SAO stroke in the univariate analysis, such as DBV, hemoglobin, and hematocrit, only DBV was still significant in the multivariate analysis adjusted for multiple vascular risk factors, including age, sex, hypertension, diabetes, hyperlipidemia, atrial fibrillation, and smoking (*p* = 0.033; Table 2). The DBV in the stroke mimic group was 240.8 ± 38.1 mP. Laboratory findings affecting blood viscosity were not different among the stroke subtypes, except for elevated D-dimer level in CE (Table 1). The baseline SBV, hematocrit, blood fibrinogen level, and BUN/Cr ratio were not different among the stroke subtypes (Fig. 3b, c, d and e).

**Table 2:** Crude and adjusted odds ratios for the association with small artery occlusion stroke

	Univariate analysis		Multivariate analysis	
	Crude OR (95% CI)	*P* value	Adjusted OR (95% CI)**	*P* value
Diastolic blood viscosity (per 1mP)	1.01 (1.00–1.02)	0.023*	1.01 (1.00–1.02)	0.033*
Systolic blood viscosity (per 1mP)	1.05 (0.99–1.12)	0.099	1.05 (0.99–1.10)	0.111
Hemoglobin (per 1 g/dL)	1.42 (1.04–1.94)	0.026*	1.27 (0.87–1.85)	0.223
Hematocrit (per 1%)	1.14 (1.01–1.28)	0.032*	1.09 (0.94–1.25)	0.252
Fibrinogen (per 1 mg/dL)	1.00 (0.99–1.01)	0.560	1.00 (0.99–1.01)	0.604
BUN/Cr ratio (per 1)	0.90 (0.80–1.01)	0.069	0.90 (0.79–1.02)	0.104

*Abbreviations*: *OR* odds ratio, *CI* confidence interval, *BUN/Cr* blood urea nitrogen-to-creatinine

*, *p* < 0.05; **, adjusted for multiple vascular risk factors including age, sex, hypertension, diabetes, hyperlipidemia, atrial fibrillation, and smoking

### Blood viscosity and chronic radiological manifestations of cerebral small vessel disease

**Fig. 4:**
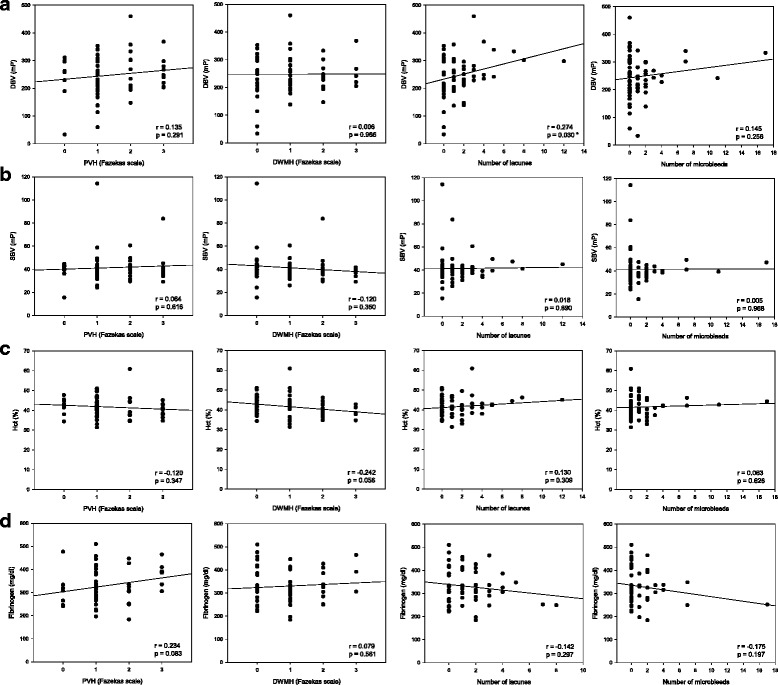
Correlation between laboratory findings, including diastolic blood viscosity **a**, systolic blood viscosity **b**, hematocrit **c**, and blood fibrinogen level **d**, and chronic radiological manifestations of cerebral small vessel disease. *, *p* <0.05; *DBV*, diastolic blood viscosity; *SBV*, systolic blood viscosity; *Hct*, hematocrit; *PVH*, periventricular hyperintensity; *DWMH*, deep white matter hyperintensity

The correlations between the major determinants of blood viscosity such as the DBV, SBV, hematocrit, and blood fibrinogen level and the chronic radiological manifestations of cerebral small vessel disease such as white matter hyperintensities, lacunes, and microbleeds, were investigated in all the patients regardless of the stroke subtype. A statistically significant correlation was observed between the DBV and the number of chronic lacunes (*r* = 0.274, *p* = 0.030; Fig. 4a). The DBV showed trends for positive correlation with the severity of PVH (*r* = 0.135, *p* = 0.291; Fig. 4a) or the number of microbleeds (*r* = 0.145, *p* = 0.256; Fig. 4a), although neither reached statistical significance. The SBV, hematocrit, and blood fibrinogen level were not significantly correlated with any of the radiological manifestations (Fig. 4b, c, and d).

### Temporal blood viscosity profiles

**Fig. 5:**
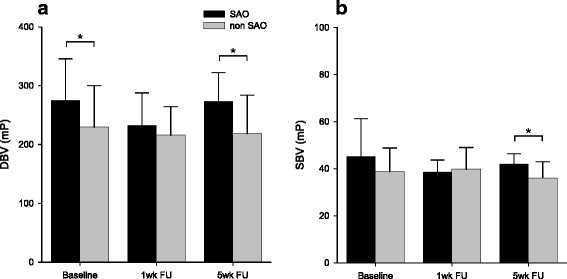
Temporal profiles of blood viscosity in SAO and non-SAO stroke. The baseline diastolic blood viscosity was higher in the SAO group than that in the non-SAO group **a**. The difference between the two groups was seen to decrease at the 1-week follow-up, and increase at the 5-week follow-up **a**. The systolic blood viscosity in the SAO group was higher than that in the non-SAO group at the 5-weeks follow-up **b**. *p < 0.05; *DBV*, diastolic blood viscosity; *SBV*, systolic blood viscosity; *SAO*, small artery occlusion

The follow-up blood viscosity was measured in 41 out of the 63 patients (65.1%). The patients who were followed up and those who were not followed up were not significantly different in their demographic characteristics (Table 3). To investigate the role of blood viscosity in SAO stroke, the patients were dichotomized into the SAO and non-SAO groups. The baseline DBV was significantly higher in the SAO group than in the non-SAO group (274.7 ± 70.9 mP and 229.8 ± 70.0 mP, respectively; *p* = 0.016; Fig. 5a). The difference between the DBVs of the two groups was seen to decrease at the 1-week follow-up (SAO, 232.7 ± 54.9 mP; non-SAO, 216.2 ± 48.0 mP; *p* = 0.394) and increase again at the 5-week follow-up (SAO, 273.2 ± 48.9 mP; non-SAO, 218.6 ± 65.4 mP; *p* = 0.009; Fig. 5a). In the SAO group, the DBV was elevated again and returned to the baseline level at the stroke onset. On the other hand, the DBV in the non-SAO group was seen to remain within the same range at the 1-week follow-up. The SBV at the 5-week follow-up was also significantly higher in the SAO group than in the non-SAO group (42.0 ± 4.3 mP and 36.1 ± 6.9 mP, respectively; *p* = 0.005; Fig. 5b).

**Table 3:** Comparison of demographics for patients followed-up and not-followed-up

	Followed-up patients (*n* = 41)	Not-followed-up patients (*n* = 22)	*P* value
Clinical findings			
Age, years (mean ± sd)	65.1 ± 13.6	67.5 ± 13.7	0.496
Male, *n* (%)	25 (61.0)	16 (72.7)	0.415
Hypertension, *n* (%)	29 (70.7)	11 (50.0)	0.169
Diabetes, *n* (%)	12 (29.3)	4 (18.2)	0.382
Hyperlipidemia, *n* (%)	6 (14.6)	4 (18.2)	0.729
Atrial fibrillation, *n* (%)	6 (14.6)	3 (13.6)	1.000
Smoking, *n* (%)	15 (36.6)	9 (40.9)	0.790
Laboratory findings (mean ± sd)			
Diastolic blood viscosity, mP	258.9 ± 72.5	226.6 ± 71.3	0.623
Systolic blood viscosity, mP	41.4 ± 9.5	41.3 ± 18.1	0.284
Hemoglobin, g/dL	14.2 ± 2.0	14.4 ± 1.7	0.598
Hematocrit, %	41.5 ± 5.5	41.9 ± 4.3	0.333
White blood cells, 10^3^/μL	7.9 ± 3.1	7.5 ± 2.2	0.133
Platelets, 10^3^/μL	228 ± 48	237 ± 73	0.104
Total protein, gm/dL	7.1 ± 0.4	6.9 ± 0.4	0.943
Fibrinogen, mg/dL	332 ± 71	325 ± 87	0.591
D-dimer, μg/dL	0.78 ± 1.25	0.63 ± 0.38	0.105
BUN/Cr ratio	21.0 ± 7.2	20.4 ± 5.0	0.500
Total cholesterol, mg/dL	167 ± 39	178 ± 36	0.943
Low-density lipoprotein, mg/dL	102 ± 34	109 ± 30	0.760
Triglycerides, mg/dL	117 ± 81	92 ± 40	0.091

*Abbreviations*: *sd* standard deviation, *BUN/Cr* blood urea nitrogen-to-creatinine

## Discussion

### Role of blood viscosity in SAO stroke

The whole blood viscosity at a low shear rate, equivalent to the DBV in our study, is dependent on the aggregation of red blood cells in the lumen of small vessels [12, 13]. Both hematocrit and fibrinogen play key roles in the aggregation of red blood cells [12, 13]. In our study, the DBV was correlated with hematocrit, while the SBV was correlated with the blood fibrinogen level. These findings appear consistent with the hypothesis that at a low shear rate, the red blood cells form a rouleaux and the blood viscosity is predominantly determined by a function of the concentration and aggregation properties of the red blood cells [24]. At a high shear rate, equivalent to the SBV in our study, the red blood cells move as free particles and the blood viscosity is determined not only by the concentration of red blood cells, but also by the plasma viscosity and deformability of red blood cells [24]. Taken together, whole blood viscosity at a low or high shear rate may be affected by the aggregation and deformability of red blood cells or plasma viscosity, respectively. In our study, we did not measure these individual parameters that contribute to whole blood viscosity. Thus, their effects on whole blood viscosity at different shear rates require further investigation.

Considering that the DBV is more important than the SBV for tissue perfusion at the level of small vessels, the DBV may be more responsible than SBV for causing cerebral small vessel disease. When the blood passes through small vessels, an elevated DBV can aggravate the flow disturbance and trigger endothelial remodeling and luminal occlusion, which can lead to acute lacunar infarction. In the general population, elevated blood viscosity at a low shear rate or plasma viscosity was correlated with silent cerebral infarction [11, 25]. In the population with stroke or at a high risk for stroke, the blood viscosity at a low shear rate and the plasma viscosity were both elevated [1]. Another study of patients with stroke showed that the blood viscosity was elevated over the entire range of shear rates, especially at low shear rates [6]. A more specific study that included patients with cerebral small vessel disease reported that the blood viscosity, plasma viscosity, and blood fibrinogen level were all elevated in patients with acute lacunar infarction [7]. More studies in relation to stroke subtypes in patients with acute ischemic stroke showed that the blood or plasma viscosity was higher in patients with SAO than in patients with LAA or CE stroke [2–4]. Consistent with previous reports suggesting hyperviscosity in SAO stroke, the DBV was higher in patients with SAO than in those with other stroke subtypes in our study. Taken together, elevated DBV at the onset of stroke can impair microvascular tissue perfusion and may be associated with the pathomechanisms involved in SAO stroke.

### Association between blood viscosity and chronic radiological manifestations of cerebral small vessel disease

The elevated blood viscosity at stroke onset may indicate not only that acute hyperviscosity triggers stroke, but also that underlying chronic hyperviscosity is present prior to stroke. White matter hyperintensities, lacunes, and microbleeds are various chronic radiological manifestations of cerebral small vessel disease [17–20]. Therefore, we hypothesized that if the blood viscosity at stroke onset is also associated with white matter hyperintensities, lacunes, or microbleeds in patients with acute ischemic stroke, the elevated blood viscosity at stroke onset might have been present prior to stroke onset.

The pathomechanisms of lacunes and white matter hyperintensities are intimately related, and acute small subcortical infarctions can progress to chronic lacunes or white matter hyperintensities [19]. Ischemia and chronic leakage of fluid and macromolecules are known to be the main pathophysiologies attributed to the development of white matter lesions [26]. More complicated pathologies of white matter lesions, including cerebral ischemia, ependymal loss, demyelination, and microcytic infarction have also been reported [17, 26, 27]. The pathophysiology of microbleeds is a leakage of red blood cells through the loosened blood-brain barrier caused by bleeding-prone small vessel disease related to hypertension or cerebral amyloid angiopathy [19]. Consistent with previous studies suggesting that blood or plasma viscosity is associated with white matter hyperintensities [11, 14, 25, 28, 29], the severity of PVH in our study showed trends for positive correlation with the DBV or blood fibrinogen level, although neither reached statistical significance. Similar to previous studies that have indicated an association between the elevated plasma viscosity and lacunar infarction [3, 11], we observed a significant positive correlation between the DBV and the number of chronic lacunes. Based on this result, the elevated blood viscosity at stroke onset may indicate hyperviscosity not only in the acute period but also in the period prior to stroke onset. A prospective observational study is needed to confirm this hypothesis.

### Recurrent blood hyperviscosity in SAO stroke

To investigate the hyperviscosity in SAO stroke, stroke subtypes were dichotomized into SAO and non-SAO groups. The DBV at stroke onset was significantly higher in the SAO group than in the non-SAO group. However, this discrepancy decreased after 1 week, as the DBV decreased more dramatically in the SAO group than in the non-SAO group. Dehydration leading to the elevation of blood viscosity is considered to be one of the major factors responsible for triggering acute ischemic stroke [3, 4, 30]. Although the DBV was higher in SAO stroke than in other stroke subtypes in our study, the BUN/Cr ratio, which generally reflects the hydration status [31], was not elevated in SAO stroke compared to that in other stroke subtypes. The elevation of DBV in SAO stroke may be caused not only by dehydration, but also by other factors determining blood viscosity. The decrease in the DBV at the 1-week follow-up may be mainly due to the hydration therapy given after admission. In the SAO group, the DBV measured at the 5-week follow-up, when the hydration therapy was terminated, showed a recurrent elevation even under the medication for secondary stroke prevention. The issue of chronic hyperviscosity after stroke has been controversial [2, 4, 5, 7, 10]. Rheological abnormalities were observed in patients even when they were under medication for secondary stroke prevention in a cross-sectional study that included patients in the chronic phase of stroke more than 3 months after the onset [5, 10]. On the contrary, acute rheological abnormalities at stroke onset normalized over 2 months in another study, regardless of the stroke subtype [2]. This discrepancy may have resulted from the different methods used to measure hyperviscosity or the different study populations. A recent serial follow-up study of blood viscosity measured at 100 s^−1^ for 2 weeks during the admission period showed that the blood viscosity was highest at the date of admission and decreased over the following 2 weeks [4]. While a serial follow-up for 2 weeks was performed during the admission period [4], the follow-up at 5 weeks in our study was performed at the outpatient clinic when the hydration therapy had already been terminated. Therefore, the results of our study may reflect the chronic status of blood viscosity after stroke, and are likely unrelated to the hydration therapy administered during admission. The recurrent elevation of the DBV in SAO stroke suggests that chronic hyperviscosity may be a persistent underlying pathomechanim of SAO stroke.

### Limitations of our study

Our study has several limitations. First, the number of patients included in our study is too small and the statistical power to support our conclusion is relatively weak. Second, a cross-sectional study design was used to investigate the role of blood viscosity in acute stroke onset or chronic radiological manifestations of cerebral small vessel disease. However, our results provide the rationale for future prospective observational studies that might confirm the cause-effect relationship. Third, follow-up of the blood viscosity was not performed in all the patients for various reasons. The number of patients lost to follow-up was relatively high (*n* = 22, 34.9%). Although there were no statistically significant differences in the baseline clinical and laboratory profiles of the patients who were followed-up and those who were not, baseline DBV showed some non-significant differences (followed-up, 258.9 ± 72.5 mP; not-followed-up, 226.6 ± 71.3 mP; *p* = 0.623). This might have led to some selection bias. Fourth, the blood viscosity can be easily influenced by hydration therapy using fluids or drinking water. In order to minimize the transient diluting effect by hydration, samples for the baseline and follow-up measurements were obtained before the hydration therapy and in a fasting state, respectively.

## Conclusions

Despite the above limitations, based on the results showing positive correlations between DBV and SAO stroke at acute stroke onset and chronic lacunar manifestation of the small vessel disease, our study suggests that elevated DBV may play a role in the development of acute and chronic manifestations of cerebral small vessel disease. The recurring elevation of the DBV in SAO stroke, even when the patients were receiving medications for secondary stroke prevention, indicates that sufficient hydration and additional therapeutic interventions targeting blood viscosity may be needed in patients with SAO stroke.

## Abbreviations

ANOVA: Analysis of variance
BUN/Cr: Blood urea nitrogen-to-creatinine
CE: Cardioembolism
Crypt: Cryptogenic stroke
DBV: Diastolic blood viscosity
DWMH: Deep white matter hyperintensity
EDTA: Ethylenediaminetetraacetic acid
FLAIR: Fluid attenuation inversion recovery
LAA: Large artery atherosclerosis
MRI: Magnetic resonance imaging
PVH: Periventricular hyperintensity
SAO: Small artery occlusion
SBV: Systolic blood viscosity
TIA: Transient ischemic attack
